# #BingeDrinking—Using Social Media to Understand College Binge Drinking: Qualitative Study

**DOI:** 10.2196/36239

**Published:** 2022-05-30

**Authors:** Madison N Cirillo, Jennifer P Halbert, Jessica Gomez Smith, Nour Sami Alamiri, Karen S Ingersoll

**Affiliations:** 1 Department of Public Health Sciences University of Virginia School of Medicine Charlottesville, VA United States; 2 Department of Psychiatry and Neurobehavioral Sciences University of Virginia School of Medicine Charlottesville, VA United States

**Keywords:** college students, binge drinking, social media, young adults

## Abstract

**Background:**

Hazardous drinking among college students persists, despite ongoing university alcohol education and alcohol intervention programs. College students often post comments or pictures of drinking episodes on social media platforms.

**Objective:**

This study aimed to understand one university’s student attitudes toward alcohol use by examining student posts about drinking on social media platforms and to identify opportunities to reduce alcohol-related harm and inform novel alcohol interventions.

**Methods:**

We analyzed social media posts from 7 social media platforms using qualitative inductive coding based on grounded theory to identify the contexts of student drinking and the attitudes and behaviors of students and peers during drinking episodes. We reviewed publicly available social media posts that referenced alcohol, collaborating with undergraduate students to select their most used platforms and develop locally relevant search terms; all posts in our data set were generated by students associated with a specific university. From the codes, we derived themes about student culture regarding alcohol use.

**Results:**

In total, 1151 social media posts were included in this study. These included 809 Twitter tweets, 113 Instagram posts, 100 Greekrank posts, 64 Reddit posts, 34 College Confidential posts, 23 Facebook posts, and 8 YouTube posts. Posts included both implicit and explicit portrayals of alcohol use. Across all types of posts reviewed, positive drinking attitudes were most common, followed by negative and then neutral attitudes, but valence varied by platform. Posts that portrayed drinking positively received positive peer feedback and indicate that drinking is viewed by students as an essential and positive part of university student culture.

**Conclusions:**

Social media provide a real-time picture of students’ behavior during their own and others’ heavy drinking. Posts portray heavy drinking as a normal part of student culture, reinforced by peers’ positive feedback on posts. Interventions for college drinking should help students manage alcohol intake in real time, provide safety information during alcohol use episodes, and raise student awareness of web-based privacy concerns and reputation management. Additional interventions for students, alumni, and parents are needed to address positive attitudes about and traditions of drinking.

## Introduction

### Background

Binge drinking among college students remains common and consequential. Approximately one-third of college students binge drink [1]. A quarter of college students report missing class, falling behind in class, doing poorly in examinations, and receiving lower grades due to drinking [2,3]. College students in the United States who drank in the last year reported episodes of forgetting where they were (28%), doing something they regretted (23%), blacking out (15%), having unprotected sex (14%), and injuring themselves (8%) [4]. Short-term severe effects of binge drinking include fatalities, motor vehicle crashes, poor academic achievement, and risky behaviors. Alcohol-related deaths per 100,000 students increased by 18% between 1998 and 2014, primarily because deaths due to alcohol poisoning doubled [5]. Nationwide, 11.2% of full-time college students reported symptoms indicating alcohol use disorder [6]. Longer-term effects of college binge drinking include unhealthy alcohol use after college, particularly among fraternity members whose frequent binge drinking continued through the age of 35 years [7]. College binge drinking remains a significant public health concern despite near-universal university interventions. Novel interventions are needed to prevent tragic outcomes during the college years and persistent problems beyond them [8-10].

**Figure 1:**
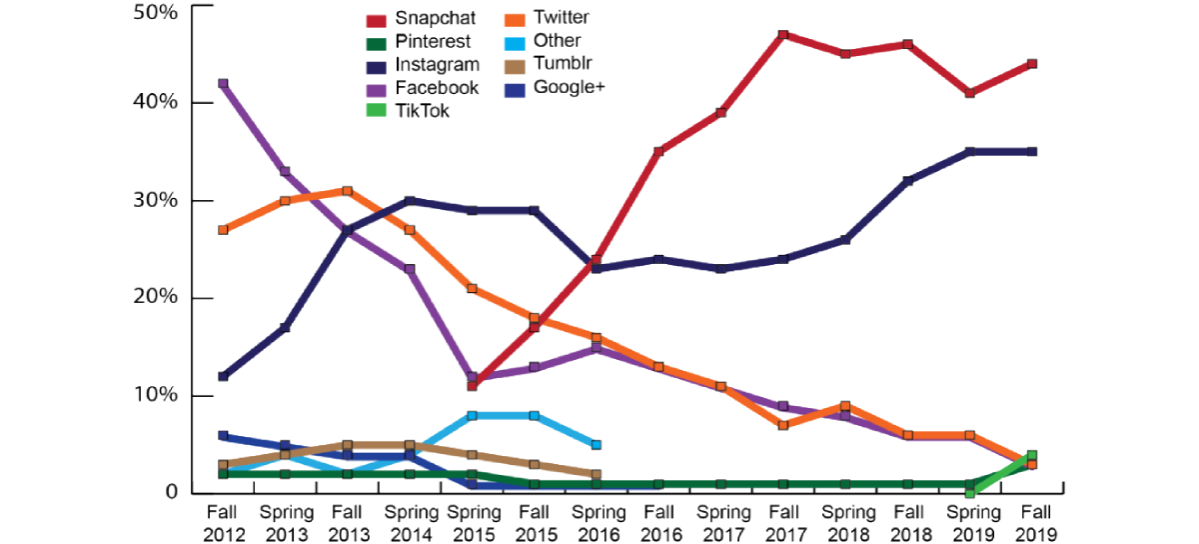
Favorite social networks of young adults 2012-2019 (based on Statista data: Piper Jaffray [19]).

Social media can provide real-time behavioral data for large populations [10,11]. For example, Twitter data have been used to track influenza symptoms, estimate alcohol sales, measure depression, track HIV prevalence, and track heart disease mortality [12-18]. Various social media platforms allow for different types of expression, and platform popularity waxes and wanes over time. However, despite the favored platform changing over time (Figure 1, based on Statista research [19]), nearly 90% of people aged 18 to 29 years use social media, with most social media users posting on multiple platforms. Among Instagram users, 90% use Facebook and 50% use Twitter concurrently [20]. Although student drinking is typically measured through surveys, social media are potentially a richer source of contextual data about drinking [21]. Autobiographical social media posts contain timely thoughts, emotions, and behaviors. Users express their identities and communicate with their peers. College students portray their current alcohol use in posts about their everyday lives, interactions with others, and engagement in activities on social media platforms.

Social media posts by college students often show risky drinking behaviors and the college drinking culture. Posts establish a web-based identity that normalizes and glamorizes binge drinking [22,23]. Most college students are exposed to peers’ alcohol-related behaviors in real life or on social media. Posting about substance use on social media is nearly universal among college students [24-26]. A study of 71 profiles found that 99% had alcohol-related content, and 10% of students posted about illicit substance use [26]. Another study analyzed *drunk* tweets and their geolocation, correlated with self-reported alcohol consumption; college towns had more tweets about excessive drinking than cities and rural areas [27].

Peer influences predict drinking among college-aged populations [28]. Viewing alcohol-related social media content is significantly associated with increased risky drinking cognition, alcohol use, and negative consequences [25,29-34]. Students usually receive positive reinforcement on the web for posting alcohol-related content [35-38]. Social validation of alcohol posts increases the number and intensity of students’ drinking behavior displays over time [10,39]. College students’ substance use posts are associated with self-reported outcomes [26,35,40,41]. Students with a web-based *drinking persona* have more motivation and intentions to drink [42], engage in hazardous drinking, and experience more alcohol-related consequences than peers without web-based drinking personas [43,44].

### Objective

Previous studies of college students’ alcohol posts have assessed single social media sites where users build social capital through networks, such as Facebook, Instagram, or Twitter. There are additional college-focused social media sites that host anonymously shared content, such as Greekrank and College Confidential. Reddit hosts *subreddits*, topic-specific discussions, including those on specific universities. This study is the first to use data from multiple social media sites to characterize alcohol-related posts among college students. Our expanded scope is important because students typically maintain multiple social media profiles [20]. The purpose of this qualitative study was to analyze social media posts about college binge drinking from multiple platforms at one university and to identify the real-time social contexts of student drinking. We hope to provide new information about behavioral targets for intervention that could inform the development of tailored mobile health interventions for college binge drinking.

## Methods

### Study Design

To develop a new intervention to prevent student binge drinking at a mid-Atlantic public university, we assessed student drinking behavior and attitudes to capture aspects of drinking culture at this particular university. This university is frequently ranked in the top 50 *party schools* in the nation [45-48], and over the last decade, it has experienced alcohol-related incidents that have created national headlines. Among several substudies to inform development of this intervention, in this study, we collected, organized, coded, and analyzed social media posts using qualitative research methods. These apply a systematic process of coding content and identifying themes and patterns into classes. The results describe the context of participants’ social media personas in a subjective but systematic scientific manner [49].

### Data Sources

Research assistants (RAs) were undergraduate and postgraduate students who captured social media posts. Social media platforms, search terms, hashtags, and specific locations for data collection were defined through iterative discussions with current undergraduates about events, traditions, and local establishments related to the drinking culture at the university. The RAs used hashtags, locations, and search terms commonly associated with the university. RAs searched social media posts from Facebook, Twitter, Instagram, YouTube, College Confidential, Greekrank, and Reddit. These platforms were selected based on the popularity of the platform among students, popularity during the timeframe under review, ability to see posts without a connection to a poster (eg, friend or follower), and durability of content (eg, content is not time limited as on a platform such as Snapchat). Each platform has varying capabilities and levels of anonymity that determine the nature of the messages produced, as described in Multimedia Appendix 1.

### Data Collection and Data Exclusion

Between March 2019 and November 2019, RAs gathered publicly available posts and comments about college students’ drinking behaviors published on 7 social media platforms between March 2013 and February 2019; all posts in our data set were generated by students associated with a specific university. RAs were assigned a site, and spreadsheets were developed with the date and time, username, gender (if known), course of study (if known), search terms, likes, shares, views, comments, and content keywords for each post. Given that we used a manual search method and manually reviewed each search term, our goal was to obtain a representative sample, acknowledging that we would not be able to find all available public posts. Instagram and Facebook required log-ins, so RAs created new accounts from which they could capture these posts. To not violate the implicit trust granted by establishing a relationship with a social media poster, these profiles were for log-in purposes only; RAs did not publish any posts, nor did they *add*, *follow*, or *friend* any accounts to view their content. RAs used school computers to search, preventing any stored identifiers on their personal devices from inadvertently influencing posts that were shown. This also prevented any bias introduced by algorithmically curated content based on user preferences. RAs copied the posts into Microsoft Excel files for text posts or saved a screen capture of multimedia posts for analysis. RAs added additional search terms based on the content found, and when finding multiple posts from one profile, we then viewed public profiles to capture additional posts. They choose posts for inclusion if they mentioned alcohol (eg, “Fireball helps me study!”), an incident known to be related to alcohol (eg, an assault on a drunk student), pictured alcohol or alcohol containers (eg, photos of discarded red solo cups outside a fraternity house), or implied alcohol use (eg, “Missed class due a late night with the boys!”). RAs assessed each post viewed using search terms published within our timeframe; however, RAs only captured posts that were relevant to alcohol use. For example, we excluded posts such as “The football team was amazing last night!” As underage drinking is an illegal activity, to protect the confidentiality of posters, all posts gathered were public, which limited the number of posts available to the research team. This prompted us to extend the original search period from 5 to 6 years. We also extended the time frame to 6 years to capture reactions to well-known alcohol-related events that occurred on campus or involved students at the university. Each post was screen captured. Images and Excel files containing written social media posts were uploaded into a qualitative analysis software (NVivo 12).

### Qualitative Coding Procedures

The data were organized using an inductive grounded theory approach [50]. Magnitude coding was used to count the number of likes received for each post on Facebook, Instagram, Twitter, and YouTube. Categories were created to display the likes each post received. Magnitude coding identified implicit and explicit drinking behavior on Facebook, Instagram, Twitter, and YouTube posts and described students’ reactions to posts on social media as positive, negative, or neutral. Open codes included concepts such as the culture surrounding alcohol use at the university, social acceptance, peer pressure, alumni influences, news surrounding tragic alcohol-related events, perceptions of Greek life, and students’ ability to drink while maintaining their academic responsibilities.

One researcher reviewed all posts and comments to design a preliminary codebook that defined the codes and described the coding procedures. Each coder (MNC and JGS) independently coded portions of the data and met weekly to update the codebook with new codes that emerged as more data were coded. An open discussion approach added a third reviewer (JPH) to discuss areas of disagreement [51,52] about how codes were applied to each social media platform. This ensured consistency in how the codes were applied but did not limit the individual coder’s view of each post in terms of positivity or explicitness. Coders recoded the data with disagreement, along with the remaining data. After the initial round of independent coding was completed, we assessed the consistency between the 2 coders using the Cohen κ score. The final κ value was 0.96, reflecting excellent interrater reliability [53]. Twitter was excluded from the overall interrater reliability owing to less than half (809/2183, 37.06%) of the total tweets collected being coded, which resulted in a low overlap of codes. Compared with Instagram, almost all posts were coded as 99% (112/113). Table 1 provides the interrater agreement for the data from each social media platform.

**Table 1:** Interrater reliability and percent agreement by social media platform.

	Cohen κ score	Percent agreement (%)
Instagram	1.00	100
Facebook	0.78	94.5
YouTube	0.86	96.3
College confidential	0.79	96.2
Greekrank	0.87	97.8
Twitter	0.42	95.6
Reddit	0.35	90.7
Overall interrater reliability	0.96	98.8

### Ethics Approval

RAs did not friend, follow, or establish a relationship with anyone who had posted; we only reviewed publicly available posts to avoid violating the privacy or implicit trust of any social media user. This study was approved by the Social and Behavioral Science Institutional Review Board of the University of Virginia (protocol 3282).

## Results

### Posts by Platform

Multimedia Appendix 1 describes the platforms, how we accessed the platform, why the platform was selected, and the number of posts coded (n=1151). Of the social media posts from 2013 to 2019 related to binge drinking at the university, Twitter generated the most posts in this study, followed by Instagram, Greekrank, and Reddit. Fewer posts were gathered from College Confidential, Facebook, and YouTube. Although we considered only using Twitter in our analysis, the other platforms offered a wide variety of viewpoints, attracted users from different perspectives, and reflected posts that may be made at different points in the drinking cycle. Owing to the team’s interest in informing intervention development, we included platforms with fewer posts to ensure that data points were from more sources in our analysis.

Social media posts by students before, during, and after alcohol use provided information about students’ and peers’ real-time attitudes and beliefs. For example, on Instagram, 2 posts illustrated student attitudes toward fake IDs. The first was a video of a creased fake ID containing personally identifiable information. The second showed a photo with the caption “three girls one fake,” clearly documenting illegal behavior by multiple students using a fake ID to obtain alcohol while under the legal age. On YouTube, a video showed a young woman looking for an entrance to the hospital emergency room encountering police, which resulted in her arrest. Many student posts included the phrase “Work hard, play hard.” Photos included a picture of a student studying for examinations, surrounded by ≥25 open cans of beer. Other posts showed alumni and parents joining in parties that included descriptions or depictions of binge drinking behavior.

Social media posts with positive reactions framed the university as a *party school*, with extreme drinking behavior as the norm. Posts on lifestyle-centered social media platforms, such as Instagram and Greekrank, tended to portray drinking in a glamourous manner, with negative comments reserved for criticizing the quality of parties and events. On platforms where users posted more about the university as an institution of higher learning (Reddit and College Confidential) than about individual student lives, we found more posts that included negative comments about the perceived drinking-centric culture at the school, implying that students who choose not to drink may have a more difficult time fitting in.

### Classification of Drinking Attitudes

Drinking attitudes on each platform were classified as positive, neutral, or negative. Table 2 summarizes the attitudes specific to each platform with exemplary posts. Table 3 reports the frequencies of each category of drinking attitudes found on each social media platform. Across all types of posts reviewed, we found that positive drinking attitudes were the most common, followed by negative and then neutral attitudes. These varied by platform. College students’ Instagram and Facebook alcohol posts frequently depicted alcohol in a positive social context (118/134, 88.1%), with fewer being classified as neutral (8/134, 6%) or negative (8/134, 6%).

**Table 2:** Definition of positive, neutral, and negative by platform.

Platform and definition			Example
**Instagram**			
	Positive	The overall tone of the user’s attitude is positive and glorifying alcohol consumption making alcohol consumption look glamorous and appealing.	Two smiling girls sitting on the steps of a deck, obviously under the influence and surrounded by red solo cups and empty bottles, with a caption about what a good day it was.
	Neutral	The user is not suggesting an opinion on alcohol. Posts that belong in this category include post about events on campus.	A photo of a historic marker near several fraternity houses, with alcohol bottles and cups in the background.
	Negative	Posts discussing the culture and behaviors of students negatively in regard to over consumption of alcohol. Posts discussing how alcohol is harmful to students and their environment.	A close-up photo of empty bottles and cups lying in the gutter of a public street with a caption about the unacceptable behavior of university students.
**Facebook**			
	Positive	The overall tone of the user’s attitude is positive and glorifying alcohol consumption making alcohol consumption look glamorous and appealing.	A photo of the membership of a fraternity outside of their house with an American flag and many of the brothers holding drinks in salute.
	Neutral	No neutral Facebook posts.	N/A^a^
	Negative	No negative Facebook posts.	N/A
**YouTube**			
	Positive	The overall tone of the user’s attitude is positive and glorifying alcohol consumption making alcohol consumption look glamorous and appealing.	A current student provides a guided tour of the collection of restaurants and bars near campus and advises on which are fun as well as easy to obtain alcohol while underage.
	Neutral	No neutral YouTube posts.	N/A
	Negative	No negative YouTube posts.	N/A
**Twitter**			
	Positive	The overall tone of the user’s attitude is positive and glorifying alcohol consumption making alcohol consumption look glamorous and appealing.	“God I love tequila and cute boys who know how to sing!” with hashtags that link it to the university.
	Neutral	The user is not suggesting an opinion, just stating a fact or news update. Posts that belong in this category include posts about traumatic events that happened.	“Alcohol and Drug Abuse Prevention Task Force challenges concept (traditions of excessive drinking by students at specific events).” (includes a link to a newspaper article that prevents several perspectives)
	Negative	Posts discussing the culture and behaviors of university students negatively in regard to over consumption of alcohol. Posts discussing how alcohol is harmful to students and their environment. Posts that belong in this category also include posts about user’s opinions on traumatic events that happened on campus.	“(Event) looks like Lily Pulitzer vomited on the entire (school) population.” “White privilege, (Event) 2017 style.” Both tweets accompanied by photos of students drinking at an event.
**College confidential**			
	Positive	Posts include students talking positively about events on campus and adjusting well into the school culture with and without consumption of alcohol.	“...but I would go again if I got to do it over (somehow I think you would not go again). A listserv primarily for notifying students of alcohol-free events was updated weekly when I attended; I can’t attest to who updates it now or how frequently. I would agree that most people drink even if they are not involved in Greek life (‘Thirsty Thursday’ is a grounds-wide saying) but I was happy enough sober.”
	Neutral	The user is not suggesting an opinion.	“Over the past couple of decades, fraternities have gradually been required to use stricter controls on parties. Guest lists, BYOB, etc. This isn’t unique to (this university) though. It’s simply a sign of the times. But it does make it harder for non-Greeks to be part of the Greek social scene. In any event, if joining the Greek system isn’t your thing, it just means you need to build your own social circle whether through clubs and whatnot. Note that after first year, a large number of university students live off campus, so I imagine a lot of Non-Greeks simply have get together at their apartments or even hang out at (local) restaurants and bars.”
	Negative	Posts discussing the culture and behaviors of university students negatively in regard to over consumption of alcohol. Students having a hard time adjusting to the culture at the university. Students complaining about excessive alcohol consumption and lack of alternative activities and ways to bond with other students.	“Bottom-line, this is NOT a good place to come if you do not plan on being a moderate to heavy party-goer. Yes, you can survive on the periphery of the social scene by not drinking but you will never get that ‘quintessential’ university experience that current students and alum rave about. Don’t let anyone try to convince you otherwise.”
**Greekrank**			
	Positive	Students rating fraternity chapters positively based on their availability of alcohol and social events. Posts in this category include posts that place higher social status on fraternity chapters based on their availability of alcohol, women, and social events.	“These guys really know how to party. Except sometimes that causes problems because I pass out at their house, but the guys are super nice and always find a way to get me home back to dorms. Great guys all the way around!” “Top house, biggest parties, coldest beers, hottest women.”
	Neutral	Posts in this category include students discussing how fraternity chapters could improve by throwing more social events and having more availability of alcohol. Fraternities in this category are average party goers. These fraternities fall somewhere in the middle between very high social status and very low.	“If you’re looking for a mild place to party, good, but if you’re looking for ragers not the place except once a year. Overall ok guys.” “Solid group of guys and definitely a top house but should probably throw more parties to remain a top house.”
	Negative	Students rating fraternity chapters negatively based on their lack of availability of alcohol, attractive women, and social events.	“A brother puked on me from above in a balcony. They have absolutely no class or alcohol tolerance. Not ‘true Southern gentlemen’ like they think they are.” “Superficial brotherhood. Serves watered down punch.” “Buncha trust fund betas who genuinely think they’re alphas.” “Drink 7 beers and pretend to black out...soft.”
**Reddit**			
	Positive	Posts include students talking positively about events on campus and adjusting well into the university culture with and without consumption of alcohol.	“You have to make your own fun. You can try to socialize with people at parties without drinking—go early before people are totally wasted. Fill that solo cup with water and laugh as people get less funny, more incoherent then leave when you’re bored.”
	Neutral	The user is not suggesting an opinion, just stating a fact or news update.	“You still need an ID at those bars unless you come in early. It doesn’t have to be a good fake but u need one.”
	Negative	Posts discussing the culture and behaviors of university students negatively in regard to over consumption of alcohol. Students complaining about excessive alcohol consumption and lack of alternative activities and ways to bond with other students.	“It is concerning that the first people encountered at the hospital did nothing to help, other than pointing them to the ER, and that it took so long after the police arrived to begin to move her into the ER—it seems this was dealt with more as a law enforcement matter than an emergency medical situation, which it could have been. That said, it is hard to feel too sorry for either one of the girls, and they should know that people who tend to become angry or combative when drunk have a strong tendency to get themselves in trouble from drinking. I seriously hope they learn something from this.”

^a^N/A: not applicable.

**Table 3:** Attitudes toward drinking by social media platform.

Types of social media		Instagram (n=111^a^), n (%)	Facebook (n=23), n (%)	Twitter, (n=373^a^) n (%)	YouTube (n=8), n (%)	Reddit (n=80^b^), n (%)	College confidential (n=38^b^), n (%)	Greekrank (n=108^b^), n (%)
**Drinking attitudes**								
	Positive	95 (85.6)	23 (100)	104 (27.9)	8 (100)	25 (39)	3 (10.7)	48 (44.4)
	Negative	8 (7.2)	0 (0)	179 (48)	0 (0)	20 (31.2)	25 (65.8)	47 (43.5)
	Neutral	8 (7.2)	0 (0)	90 (24.1)	0 (0)	35 (54.6)	10 (26.3)	13 (12)

^a^Not all posts were directly related to drinking attitudes and only those posts that displayed a drinking attitude were included.

^b^This platform included posts that displayed multiple drinking attitudes.

### Classification of Alcohol Use Depictions

Drinking experiences were classified as implicit or explicit on platforms with photographs. Explicit alcohol use was common on Instagram and Facebook, seen in 66.7% (74/111) of Instagram posts, whereas the remaining Instagram posts alluded to alcohol consumption and were coded as implicit (37/111, 33.3%). Facebook posts were usually explicit in depicting alcohol use (21/23, 91%), with a few implicit consumption posts (2/23, 9%). Facebook, Instagram, and YouTube used a visual medium and were grouped together for the analysis. Posts on these platforms (with similar definitions of positive, neutral, and negative) showed more explicit alcohol consumption (102/140, 72.9%; Table 2). Nearly all posts containing explicit alcohol consumption were depicted as positive by the person posting (99/102, 97%); only 3% (3/102) were portrayed as negative. Similarly, implicit alcohol consumption was mostly portrayed as positive (31/38, 81%), with only 18% (7/38) of these posts seemingly negative by the person posting. All YouTube posts were categorized as positive and explicit.

In contrast, posts on Reddit, College Confidential, and Greekrank showed a wider range of views on alcohol events. Overall, 31% (20/64) of the Reddit posts portrayed alcohol consumption as negative, 54% (35/64) as neutral, and 39% (25/64) as positive. College Confidential frequently portrayed alcohol consumption as negative (25/34, 65%), with a minority of posts about alcohol (3/34, 10%) being positive. Greekrank posts were evenly split as positive (48/71, 44%) or negative (47/71, 43%). Compared with Instagram, Facebook, and YouTube, College Confidential, Greekrank, and Reddit had fewer alcohol-related posts depicted as positive. Finally, we identified high-risk drinking events in the local areas where college students tend to frequently binge drink. Examples included gatherings at local bars and restaurants, festivals, fraternity houses, yearly events, and traditions around academic breaks, seasons of the year, and sports events. Most of these events were displayed positively. Negative events (eg, student injuries) were positive toward the student but negative toward the university administration. Of the posts that exhibited high-risk drinking events, most (274/370, 74.1%) were shown in a positive social context, whereas only 25.9% (96/370) were shown in a negative social context.

### Peer Reactions to Posts

On average, Instagram posts received 350 *likes*, or positive feedback tags placed on posts by viewers other than the original poster to indicate their reaction (mean 350.1, SD 1224.38). More than half of all Instagram posts had over 100 likes (65/111, 58.6%). On Instagram, only 12.6% (14/111) of the posts had fewer than 20 likes. Facebook posts received 43 likes on average (mean 43.3, SD 51.91), and only 17% (4/23) of Facebook posts had over 100 likes, whereas nearly half of all Facebook posts had fewer than 20 likes for each post (11/23, 48%). Table 4 summarizes peer feedback on these posts.

**Table 4:** Implicit and explicit alcohol content on Instagram, Facebook, and YouTube.

Drinking behaviors		Instagram (n=111), n (%)	Facebook (n=23), n (%)	YouTube (n=8), n (%)
Implicit alcohol content		37 (33.3)	2 (8.7)	0 (0)
Explicit alcohol content		74 (66.7)	21 (91.3)	8 (100)
**Count of “likes” (if videos, count of “views”)**				
	<20	14 (12.6)	11 (47.8)	0 (0)
	20-50	15 (13.5)	6 (26.1)	0 (0)
	50-100	17 (15.3)	2 (8.7)	1 (12.5)
	100-500	51 (45.9)	4 (17.4)	1 (12.5)
	≥500	14 (12.6)	0 (0)	6 (75)

### Common Themes

As we worked with the data, the coders noticed that certain words frequently appeared. The largest words showed the highest frequency, included alcohol, culture, Greek, drunk, and party. The visual representation of the word cloud shows the common words in the content of social media posts.

## Discussion

### Principal Findings

Students post alcohol use content, which was then liked, retweeted, and shared, likely reinforcing the poster, who is creating a public image of a desirable life to their peers. Students freely shared their drinking behaviors and attitudes toward alcohol use on publicly accessible social media platforms [54,55], visible not only by peers but also by school administrators, parents, and future employers. Thus, the impact of student posts could extend beyond current social networks, but posts reflected little concern about how social media history could influence future prospects.

Alcohol-related events including student deaths have made headlines. Some social media posts showed that students believed that the university should have done more to prevent these tragedies. In other cases, social media posts indicated an attitude of blaming the victims for being in these dangerous situations. Few posts mentioned the dangers related to excessive drinking or taking responsibility for drinking behavior. Instead, more posts showed students in situations where they could use help and guidance, such as posts showing students passed out in public spaces, intoxicated while walking on the streets, and other situations where their safety and well-being are at risk. These posts demonstrate that students may need a convenient, easy-to-understand resource that explains the university’s policies on alcohol use and how to obtain help. This guidance should be available at the moment of excessive drinking.

Several social media posts included multiple generations of alumni and current students drinking alcohol together. These posts demonstrate that drinking is normative and multigenerational. This implies that students must drink alcohol to have a typical, happy experience at the university. Although there were some posts critical of the portrayed drinking culture, a few posts showed that moderate drinking is an option. A few posts shared the idea that it is possible to have a satisfying, fun, and enjoyable college social life without participating in binge drinking. Some students indicated a desire for greater availability of events and opportunities that are not centered on alcohol use, both by students who abstain and those who would like a break from always drinking when socializing.

### Limitations

We did not capture every social media post on the selected platforms nor did we capture data from all possible social media platforms. For example, Snapchat is a popular platform in which posts are time limited, with privacy protections that limit the public availability of posts, making it unavailable for data collection. Snapchat videos, also known as *stories*, were not investigated, as they cannot be seen unless you are a friend of the poster, which adds a limitation. Stories are deleted after 24 hours, and it is possible that it is a key platform for sharing binge drinking [54] that we were unable to access. Platforms such as Facebook and Instagram have increased privacy options, and users may restrict nonfollowers from viewing their content. By limiting the searches for this study to publicly available posts, we may have missed content that portrays different drinking norms. In addition, searching platforms such as Twitter is complicated, as hashtag searches often yield numerous posts that are not relevant to the topic. For example, searching for the university name resulted in posts about sports teams, faculty news, and other non–student-generated content. Despite these limitations, we captured a representative sample of public student posts from different platforms. The student perspectives expressed on publicly available social media show little evidence of *faking good* that could occur when participants are interviewed or surveyed.

### Comparison With Prior Work

Other studies on social media and binge drinking have examined responses to specific messages [56,57], the number of posts [58], and habits of social media use [58,59] compared with reported drinking behavior. This study adds to the literature by analyzing the content of posts and responses to posts from multiple platforms. These data create a picture of drinking behaviors at the university that has implications for the development of interventions. Specifically, this study identified 5 concerning themes that the interventions could address. First, public posts clearly showed students engaging in risky drinking behaviors. Tips and tools for managing drinking (and posting about it) in real time are needed. Second, several posts showed that students who did not know how to access or ask for help because of concerns that they or their friends would face consequences. Third, social media posts depict a norm that all students at the university drink heavily and that a happy social life depends upon binge drinking. Fourth, these public posts could sometimes harm student reputation. Fifth, several posts showed that parents and alumni were part of the drinking culture, including posts made by parents while drinking with students.

On the basis of these themes, we offer 5 recommendations to reduce harm related to excessive drinking among college students. First, the early timing (often before or during the first semester of college) and universal targets of alcohol education should be reconsidered. In addition to existing alcohol education, we assert that there is a need for tailored, easy-to-use tools that students can use in real time when drinking. Second, universities should show students how to obtain help from themselves or their peers during drinking without penalty, even if they are underage. Third, students at the university (and likely others) need assistance in finding popular alcohol-free social activities. Fourth, colleges should raise students’ awareness of their web-based reputations and provide options to help them repair their web-based reputations if public posts show them under the influence of alcohol. Fifth, universities should encourage parents and alumni to modify their own drinking habits at university events or gatherings to provide better role modeling. Previous studies have found that interventions encouraging parents to model acceptable limits for alcohol consumption can have a positive impact on delaying and reducing student drinking [60-64].

### Conclusions

This qualitative analysis of social media posts on college student drinking is the first to characterize student posting and commenting behavior across multiple social media platforms. This adds to a growing body of literature showing that analyzing social media can reveal the context of hazardous drinking behavior [65]. An important contribution of this study is that it demonstrated the attitudes and actions of students during binge drinking; these may differ substantially from what students report on alcohol use surveys. Important and novel findings are as follows: (1) social media platforms are being used before, during, and after the time of hazardous drinking; (2) most posts showing consequences of excessive drinking occurred in near real time; (3) the majority of posts showing explicit alcohol consumption were positively reciprocated with more likes and comments indicating students’ positive attitudes toward risky drinking behaviors; peers’ comments about their peers’ posts create an web-based social context that strongly reinforces risky drinking behavior; and (4) students are often depicted in risky situations when drinking, and posts about these could damage student reputations or future prospects. The study identified students’ thoughts and beliefs about the binge drinking culture at one university, but it is likely that the concerning themes and resulting recommendations will be generalized to other colleges.

Finally, this social media review identifies several new targets for intervention. Students need real-time interventions during their drinking episodes before they experience harm. Students lack awareness of resources for improving safety for themselves and their peers while drinking and need to access this guidance when witnessing excess drinking. Students also need consciousness-raising interventions regarding the risks of creating or allowing social media posts during drinking episodes. These and other behavioral targets could be addressed by platforms such as mobile apps that could provide information during drinking episodes. A thoughtfully developed mobile app could provide tailored, real-time tracking of drinking behavior; guidance to improve student drinking safety; and reminders against posting content that could harm reputations and limit future options.

## Appendix

Multimedia Appendix 1 Social media platform descriptions, search methods, restrictions, and yield for the study.

## Abbreviations

RA: research assistant
