# Supplementary material for: #BingeDrinking—Using Social Media to Understand College Binge Drinking: Qualitative Study
Source: JMIR Hum Factors. 2022 May 30;9(2):e36239. doi: 10.2196/36239 (PMC9153908; doi:10.2196/36239)
Supplement: Multimedia Appendix 1 [file humanfactors_v9i2e36239_app1.docx]

| Platform | Description and search methods | Account required for data collection? | Number of posts |
| --- | --- | --- | --- |
| Instagram | - Instagram is photo sharing site that allows both public and private sharing. - Users must have an Instagram account to view and comment on other users’ posts. - Data collected were gathered by typing terms and post locations into the search tool bar to review for relevant posts. | Yes | 113 |
| Facebook | - Facebook profiles share text, pictures, music, videos, links, and other content with users they both know and do not know. - No users were friended to access their posts, so Facebook posts were limited to publicly available profiles. - Forty-five percent of users nationwide reported that their social media accounts are private. Nineteen percent stated that none of their social media accounts are private (Clement, 2018). | Yes | 23 |
| YouTube | - YouTube is a video sharing site where users post videos and comment on other users’ content. - Posts were collected by using preset search terms to find relevant videos. - Ninety percent of American aged 18-24 years report using YouTube (Perrin & Anderson, 2019). - A total of 8 posts were included in the coding analysis. Of 8 YouTube videos, 5 had over 1000 views. | No | 8 |
| Twitter | - Twitter is a microblogging site where users send and receive short posts called tweets. - Original tweets included up to 140 characters and links to other web-based content. Tweets now include up to 280 characters, 4 photos, 1 GIF or video. - Twitter posts were collected manually with preset hashtags and search terms. - Reviewers converted results pages for every search term and hashtag into a PDF file and uploaded to NVivo 12. Coders reviewed each PDF file and coded relevant tweets and photos. - 64 PDF documents captured 2183 tweets, including content not relevant to the study (eg, comments about basketball team, a street name identical to one at the campus but in Australia). Coders performed the *exclusion* review while collecting posts on other platforms. We only included coded posts in our final sample. | No | 809 coded tweets in 64 PDFs |
| College confidential | - Large college-related messaging board on different colleges and universities. - Site is popular as students make decisions about which colleges are a good fit academically and socially during applications. - All posts are anonymous. - Users create posts and can comment on other users’ posts. - Posts collected by specifying the university and preset terms in the search tool bar. - Data collection was complete when all search terms were used to find relevant posts. | No | 34 |
| Reddit | - Reddit is a collection of web-based discussion forums that discuss specific topics where users share news, content, and links related to the topics. - Each forum is moderated by specific users who can delete or block content. - Reddit users can create anonymous posts and comment on other users’ submissions. - Significant posts were gathered by typing terms into the search tool bar and reviewing results. - Once all search terms were used to identify posts, data collection was complete. | No | 64 |
| Greekrank | - Users post about their experiences at Greek organizations, including rushing, membership, formals, and events. - Each Greek organization is rated on 7 criteria (friendliness, popularity, campus involvement, classiness, fun-social life, brotherhood, and tier). - Once all search terms had been reviewed for relevant posts, data collection was complete. - Research shows that college students who belong to social fraternities or sororities have considerably higher rates of substance use than their college peers who do not join such organizations (Maggs et al, 2011; Glassman et al, 2010; Hutching et al, 2008; DeSimone et al 2007). - Included because 35% of this university’s students are involved in Greek life. | No | 100 |
